# Authenticity: Effective emotional labor strategies on teaching efficacy of university teachers in China

**DOI:** 10.1371/journal.pone.0297760

**Published:** 2024-01-26

**Authors:** Jiuhua Zheng, Yuqing Geng, Juan Gao, Qinjun Xiang

**Affiliations:** 1 College of Economics and Management, Shanghai Ocean University, Shanghai, China; 2 School of Business, Shanghai Dianji University, Shanghai, China; Guangxi Normal University, CHINA

## Abstract

Based on the job demands-resources model, this study explored the relationships among emotional labor strategies, job demands of teaching, job resources, and teaching efficacy for university teachers. The results, based on a sample of 316 university teachers from China, showed that the teachers preferred to convey authenticity by expressing deep acting and naturally felt emotion. Furthermore, deep acting and naturally felt emotions were found to have a significant positive effect on teaching efficacy, whereas surface acting did not have any significant relationship with teaching efficacy. As organizational variables, job demands significantly positively affected surface acting, deep acting, and naturally felt emotion. In contrast, job resources positively affected surface and deep acting, but no significant relationship with naturally felt emotions was found. These results highlight that university teachers from China could benefit from adopting authentic emotional labor strategies, specifically deep acting and naturally felt emotions, as the most effective strategies in teaching. Based on the above findings, this paper concludes with recommendations for university administrators to alleviate the emotional labor of college faculty. For example, universities should pay more attention to teachers’ emotional state, provide resources to support them, and give more space and support to college teachers in teaching.

## 1. Introduction

Since the introduction of the concept of emotional labor by Hochschild [1], research on emotional labor in education has received increasing attention. It is widely accepted that schools are settings that require emotional labor, with teachers’ work being rife with various forms of emotional labor [2]. Within the realm of higher education, emotions are recognized as playing a crucial role in effective teaching [3], and teachers consider the expression of emotions in front of students as a skill and qualified teachers are known to employ emotional management skills during their teaching effectively [4]. Another study built on Morris and Feldman’s work and proposed the concept of emotional labor in teaching, which refers to the effort, planning, and control required for teachers to express organizationally desired emotions during their interpersonal transactions with students and others in classroom and school settings [5]. Meanwhile, according to Hochschild’s (1983) criteria, university teachers’ teaching process is emotional labor [6, 7].

Despite the growing recognition of teachers’ emotional labor in recent years, research focusing on teaching-related emotional labor still needs to be completed. Previous studies have predominantly focused on analyzing the dimensions and magnitude of emotional labor [8, 9], as well as antecedent variables such as work pressure [10], and outcome variables such as burnout [11, 12], job satisfaction [13, 14], and well-being [15]. However, these studies have mainly focused on individual teachers, leaving little knowledge about the relationship between teachers’ emotional labor and teaching efficacy, a key performance indicator. Moreover, most research subjects are primary and secondary school teachers [2, 5] and university administrative staff members [16]. As university teachers differ from primary and secondary school teachers and traditional service industries, the emotional labor involved in their work is more complex due to the nature of the objects involved, demanding a heightened authenticity in their engagement with students. Concerning emotional labor, authenticity, not fake, is attained through deep acting and genuine emotional expressions rather than surface acting [17, 18]. Nevertheless, the effects of emotional labor on university teachers remain unexplored and require further investigation [19].

Job demands and resources can influence the emotional labor strategies of university teachers. In order to comply with the expectations of the organization, teachers need to express the desired emotional state [20], which can be influenced by the demands of universities and the traditional cultural expectations of the teacher’s role. Universities’ demands are often characterized as emotional rules [21, 22] or rules of performance [23], which can implicitly or explicitly inform teachers which emotions and expressions are appropriate to convey, particularly in interactions with students. The research indicates significant differences between public and private universities [24, 25], but in this paper, it only refers to public universities. Teachers must regulate their emotional expressions in teaching to meet the demands of their university and occupation, which can lead to burnout. To minimize the harmful effects of job demands, universities can provide resource support to reduce teachers’ self-destruction and improve their emotional regulation skills [26, 27]. Such actions benefit higher education growth and labor performances with sustainability [28–30]. Nonetheless, it remains unclear how these dual processes affect emotional labor strategies.

To gain a deeper comprehension of the impact of emotional labor on university teaching and to bridge the gaps in current literature, this study employs the job demands-resources model to explore the correlation between emotional labor and teaching efficacy in organizational settings via quantitative research. The outcomes of this research endeavor have the potential to furnish fresh evidence that contributes to a more comprehensive understanding of the effects of organizational factors on the emotional labor of college educators and their consequent job efficacy. Moreover, the study intends to provide suggestions and references for university administrators to optimize their practices and policies.

## 2. Literature and hypotheses

### 2.1 Job demands-resources model

Demerouti et al. have categorized job characteristics into two overarching categories: job demands and resources [31]. These categories have a varying impact on employees, depending on whether they contribute to exhaustion or gain. Job demands are associated with the depletion of an employee’s resources, as the job requires sustained physical or mental effort, leading to physiological and psychological costs. The negative impact of job demands is evident in employees’ physical and mental health, with higher demands leading to job burnout, fatigue, and even job loss [32]. In contrast, job resources refer to the resources the organization provides to support employees in achieving their work goals, reduce their physiological and psychological costs, and stimulate their personal growth and development, such as organizational support, harmonious relationship with colleagues, and work control. However, due to inter-individual differences, such as self-efficacy, employees’ perceptions of job demands and resources may differ under similar work conditions [33].

The study of emotional labor can be approached in two fundamental ways: job-focused and employee-focused [34]. Both approaches acknowledge that emotional labor significantly impacts employee efficacy, but they differ in their focus. The job-focused approach concentrates on the demands of emotional expression at the organizational level, including expression rules, frequency, and diversity of specific emotional expressions. On the other hand, the employee-focused approach focuses on the emotion regulation process at the individual level to meet job demands. However, the job demands-resources model combines both research approaches and provides a comprehensive picture of the mechanisms that describe the role of emotional labor in the workplace. Empirical research in organizational and occupational psychology has supported the applicability of this model [32, 35]. While previous studies on teachers’ emotional labor have examined only a subset of the relationships presented in the job demands-resources model, such as the relationships between teachers’ emotional labor and emotional job demands [36], job resources [37], and social support [38, 39], few studies have comprehensively applied the model to explore the underlying processes in university teachers’ emotional labor. Therefore, a significant gap in the literature needs to be addressed by comprehensive research that applies the job demands-resources model to investigate the relationships between emotional labor and teaching efficacy in university settings.

### 2.2 Emotional job demands of teaching and university teachers’ emotional labor

Changes in job demands in the teaching field necessitate university teachers to carry out emotional labor. The job demands in teaching refer to the rules and expectations of organizations or managers regarding the display of emotions by employees [40]. Continuous reforms in higher education have led to higher demands on university teachers, not only in terms of increased class hours but also in terms of using high levels of emotions to enhance the quality of teaching, which has been confirmed by many studies [6, 13]. Teachers in higher education are subjected to various demands in their work that subsequently affect their emotions when interacting with students [41]. University teachers’ teaching duties encompass classroom teaching time and activities outside the classroom [42]. Consequently, teachers may need to engage in emotional labor when interacting with students, both in and out of class. In recent years, the "student-centered" teaching method has gained popularity, requiring teachers to take up additional teaching roles besides classroom teaching, such as course tutors and project supervisors; such frequent and intense teaching work necessitates that teachers manage their emotions effectively. Besides, constructing a harmonious teacher-student relationship has become a mainstream practice, eliciting explicit and implicit demands to display teachers’ emotions [7]. Exact demands include the frequency and duration of teacher-student interactions, such as regular student communication. In contrast, implicit demands may require teachers to exhibit positive emotions, such as kindness and affinity towards students.

In traditional Chinese culture, social expectations trigger self-imposed demands among teachers. Teachers are respected throughout the ages and viewed as the most honorable profession, defined as those who preach and teach. Deeply rooted concepts, such as "a teacher for a day is a father for a lifetime" and "respecting teachers and loving students," require teachers to care about students genuinely. Similarly, "What is a father? A good teacher. What is a teacher? A strict preacher" emphasizes the teacher’s responsibility for students’ learning outcomes while highlighting their concern for students. In addition to their professional authority, university teachers play the roles of teacher, parent, and authority in teaching [5], reinforcing the cultural expectation to demand and restrict themselves. Accordingly, emotions are regulated according to cultural expectations, not solely for financial compensation, but because it is considered appropriate and necessary to do so [7]. The results of previous studies have shown findings that teachers scored higher on deep acting compared to surface acting [5, 43], and Li et al.’s study showed that in Chinese culture, teachers use deep acting and expression of naturally felt emotions more often and surface acting less often [44]. Considering teachers’ roles as parents and cultural preferences for authentic emotional expression, this study proposes that university teachers prefer deep acting and naturally felt emotions over surface acting.

**H1:** University teachers prefer deep acting and naturally felt emotions over surface acting.

The emotional demands of teaching have different effects on emotional labor strategies. The perception of display rules is influenced by the explicit or implicit expectations of the organization [45], while university teachers’ understanding of these rules is subjective [46]. According to the job demands-resources model, job demands are critical organizational factors that can prompt teachers to engage in emotion management, and research in this area has focused on job demands. Morris and Feldman (1996) have proposed that emotional labor originates from emotional demands that express organizational expectations in interpersonal communication. Such emotional demands are rooted in organizational and cultural teaching expectations and can motivate teachers to assess the demands of their job and adopt different emotional labor strategies. Teachers may be required to engage in surface and deep acting to meet organizational demands by altering their appearance or cognitive state. Studies by Yin et al. (2017) and Zheng et al. (2022) have demonstrated a positive correlation between teachers’ perceived emotional labor demands and their use of surface and deep acting. However, naturally felt emotions that come from the heart do not require effort from the teacher, so the job demands may impair the expression of such emotions, resulting in a negative correlation. Based on these findings, we propose the following hypotheses.

**H2a:** Emotional job demands of university teaching are positively related to surface acting.
**H2b:** Emotional job demands of university teaching are positively related to deep acting.
**H2c:** Emotional job demands of university teaching are negatively related to the expression of naturally felt emotions.

### 2.3 Job resources of teaching and university teachers’ emotional labor

According to the job demands-resources model, the job resources provided by the organization will also impact emotional labor strategies. "Job resources" are the material and psychological resources provided by the organization; a lack of job resources will lead to burnout and reduce the sense of accomplishment at work. In contrast, providing job resources can cushion the pressure brought by job demands, reduce the consumption of emotion [47], weaken the psychological and physical attrition caused by job demands, and promote smoother work. Among the organization’s resources, the most basic and essential resource is the organizational support perceived by employees.

Organizational support affects the emotional labor strategies adopted by university faculty. Perceived organizational support is the degree to which employees perceive that the organization cares about their contributions and well-being, and thus the feelings and perceptions they form about organizational support [48]. According to organizational support theory, organizational support can give employees positive emotional support, stimulate work enthusiasm and motivation, and positively affect employees’ positive behaviors, thus improving work performance [49]. As a job resource, some studies proved that organizational support can reduce negative emotions caused by stress [50, 51]. Emotional labor is also used in classroom teaching in universities that require long, unpredictable, and emotional displays that may require exaggerating of some emotions. Emotional labor will also stimulate and cultivate university students’ thoughts and interactions in off-course counseling and tutoring. In order to buffer university teachers from emotional labor, universities provide a supportive working environment that gives them respite from the needs of their students [52]. According to the principle of reciprocity, university teachers who receive support will work harder following their job demands to repay their support. Wu et al. (2020) also argue that lower levels of peer and mentor support are associated with higher levels of emotional labor in academic settings [39]. Therefore, when given high resources, university teachers will strive to achieve the organization’s demands and adopt deep acting. When resources are insufficient, surface acting is usually used to meet the job demands.

Organizational support will enable university teachers to treat colleagues and students authentically. In recent years, the continuous reform of universities in China requires teachers to treat students sincerely and care about them, regardless of relevant policies and public opinion advocacy. It also provides a series of support. In this culture, when students misbehave, they are usually considered careless rather than intentional. As long as teachers are acting for the good of their students, both positive and negative emotions will be accepted, and even the appropriate display of negative emotions to be strict with students will be interpreted as a sign of responsibility for them [5], this will encourage university teachers to share their true feelings. Therefore, the higher the organizational support, the more university teachers can authenticate their emotions. Hypotheses are as follows:

**H3a:** The perceived job resources negatively relate to surface acting.
**H3b:** The perceived job resources positively relate to deep acting.
**H3c:** The perceived job resources positively relate to the expression of naturally felt emotions.

### 2.4 Emotional labor and teaching efficacy of university teachers

Bandura’s self-efficacy theory posits that an individual’s beliefs about their confidence and success in a specific environment are influenced by their perceived ability to meet organizational demands and perform effectively [53]. Teaching efficacy refers to a teacher’s judgment and feelings about their ability to teach and positively impact students’ learning outcomes and behaviors. Tschannen-Moran and Hoy define "teaching efficacy" as a teacher’s judgment of their ability to achieve desired student engagement and learning outcomes [54]. They also have proposed measuring it regarding instructional strategies, class management, and student learning engagement. Chang et al. integrated Tschannen-Moran and Hoy’s (2001) definition [55]. Further, they expanded the concept of teaching efficacy in terms of course design, instructional strategies, technology usage, class management, interpersonal relations, and learning assessment within the context of Chinese culture. Bandura’s theory also suggests that teachers’ perceptions of the teaching environment and feedback and support from administrators are influential factors in determining teaching efficacy and various performances.

Ashforth and Humphrey (1993) identified the potential relationship between emotional labor and self-efficacy, which prompted research in this area [23]. However, the majority of research in the decades since has focused on emotional labor and well-being-related variables, such as burnout or job satisfaction, rather than job performance-related variables, such as teaching efficacy. In the field of education, similar trends are observed, with less research on teaching efficacy. Nevertheless, teaching efficacy is crucial to teaching outcomes. It has been shown to have significant effects on job burnout [56], job satisfaction [57], organizational commitment [58], and turnover intention [59] and other variables among teachers, and is closely related to their emotional fluctuations [60]. Furthermore, teaching efficacy impacts students’ academic achievement [61]. Therefore, understanding university teachers’ teaching efficacy is paramount for effective teaching outcomes.

Broadly, teaching efficacy is a sense of personal accomplishment; in the context of emotional labor, surface acting is typically associated with low self-efficacy, as an individual’s emotions fail to meet the demands and expectations of the job. Conversely, deep acting indicates high self-efficacy, as it successfully changes perceptions or cognition to display the emotions required by the job [34]. Some studies suggest that authenticity, including deep acting and the expression of naturally felt emotions, can enhance self-efficacy and reduce the adoption of surface acting [5, 15]. However, other studies, such as Xie et al., indicate that teachers’ naturally felt emotions and surface acting can improve teaching efficacy [62]. Insufficient studies have been conducted on university teachers [63], and further research is needed to explore the relationship between emotional labor and teaching efficacy in this group. Based on the job demands-resources model, emotional labor may have positive and negative effects on university teachers’ teaching efficacy, which can be reduced by surface acting and increased by deep acting and naturally felt emotions. Whether emotional labor positively or negatively affects teaching efficacy depends on how university teachers perceive job demands and resources and whether they adopt surface-acting or authentic deep-acting and naturally felt emotions. Therefore, the following hypotheses are proposed.

**H4a:** Surface acting negatively relates to teaching efficacy in instructional strategy, class management, and interpersonal relation.
**H4b:** Deep acting positively relates to teaching efficacy in instructional strategy, class management, and interpersonal relation.
**H4c:** Expression of naturally felt emotions positively relates to teaching efficacy in instructional strategy, class management, and interpersonal relation.

**Fig 1:**
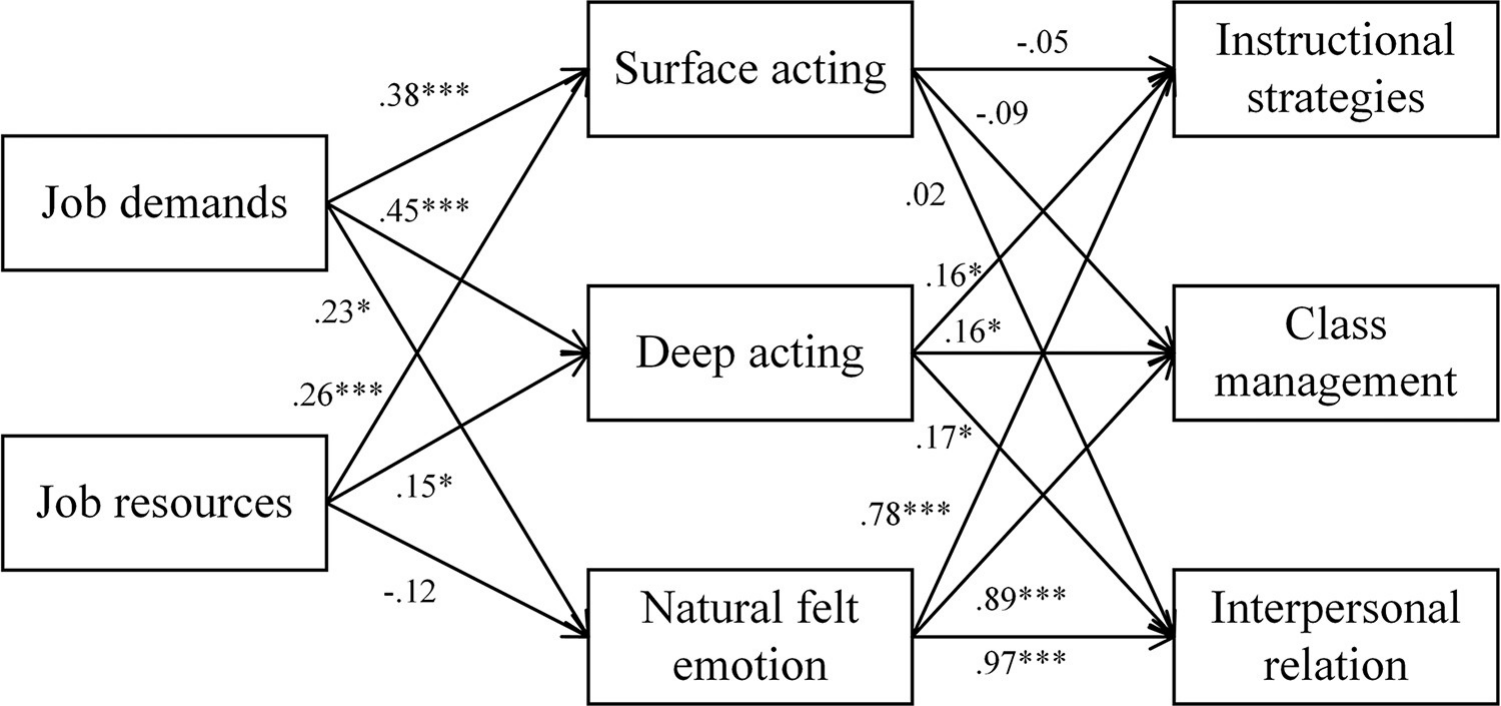
The hypothesized model of the study.

The model of this study is constructed based on the hypotheses presented above, as shown in Fig 1.

## 3. Method

### 3.1 Participants and procedures

During the epidemic control period, data was collected through on-site questionnaire distribution and collection for local participants in Shanghai, while online responses were used for data collection from teachers in other regions. After validation of the online responses, red packets were given to the participants. 342 questionnaires were distributed nationwide, and 316 valid questionnaires were returned, resulting in a response rate of 92.40%. Of the 316 questionnaires, 66.1% were female, and 33.9% were male. The age ranged from 23 to 65, with an average age of 41.23 (SD = 7.15) years. Participants had teaching experiences ranging from 1–42 years, with an average teaching experience of 14.28 (SD = 8.73). Among the participants, 10.8% held the title of professor, 41.5% were associate professors, 41.8% were lecturers, and 5.9% were teaching assistants. Regarding educational attainment, 57.0% held a doctoral degree, 38.9% held a master’s degree, and 4.1% held a bachelor’s degree. The participants were from various university categories, with 8.3% from "985" Universities, 14.2% from "211" Universities, 70.9% from regular universities, and 6.6% from specialist universities. The participants also represented various disciplines, with 35.76% from science and engineering and 64.24% from humanities disciplines. On average, participants reported teaching 8.20 (SD = 4.34) weekly credit hours.

This research is exempt from the Academic Ethics Committee’s review according to the regulations of authors’ affiliations, which are certified by the committee. Furthermore, we obtained verbal consent from human participants before distributing questionnaires. At the beginning of the questionnaires, we clearly explained the purpose and content of the study to the participants and showed them the consent form; they could fill in the questionnaires only after they agreed. The recruitment period for this study is from September to October 2022.

### 3.2 Instruments

#### Job demands

This study adopted the Perceived Display Rule Demands developed by Diefendorff et al. (2005) [40], with seven items, including four positive display rule perceptions, such as "my organization expects me to try to act excited and enthusiastic in my interactions with students" and three negative display rule perceptions, such as "this organization expects me to try to pretend that I am not upset or distressed. " The items were scored from 1 (strongly disagree) to 5 (strongly agree). The Cronbach’s αin this study was 0.91.

#### Job resource

This study adopted peer support and administrative support of Perceived Teaching Support designed by Chang et al. (2010) [55]; each dimension has five items, such as" the administrators are concerned whether the teaching load is manageable." The items were scored from 1 (strongly disagree) to 5 (strongly agree). The Cronbach’s αin this study was 0.91.

#### Emotional labor strategy scale

The 12-item Emotional Labor Strategy Scale, adapted from Diefendorff et al. (2005) and validated by Min-er [64], was used to measure teachers’ emotional labor strategy. It is divided into three dimensions, including six items on the surface acting, such as "I fake the emotions I show when dealing with students," 4 items on the deep acting, such as "I try actually to experience the emotions that I must show to customers "; and two items on the naturally felt emotions, such as "the emotions I express to students are genuine." The items were scored from 1 (strongly disagree) to 5 (strongly agree). The Cronbach’s αin this study was 0.76.

#### Teaching efficacy

This study used three dimensions of instructional strategy, class management, and interpersonal relation from the Teaching Efficacy Scale for College Teachers developed by Chang et al. (2011) [65]. The scale consists of 13 questions, with five for all dimensions except for interpersonal relation, such as "have confidence in inspiring and maintaining students’ learning motivation. " The items were scored from 1 (strongly disagree) to 5 (strongly agree). The Cronbach’s αin this study was 0.95.

### 3.3 Analyses

The initial analysis of the data involved an examination of missing values, revealing that no variable had more than 5% missing values. To replace the missing data, we utilized the mean substitution method. Descriptive statistics, reliability, and other indicators were analyzed using SPSS Ver. 25 software. Additionally, hypothesis testing of the model was conducted using Amos Ver. 22 software.

## 4. Results

### 4.1 Common method variance

The data in this study were obtained from self-reports, so Harman’s single-factor test was used to examine common method bias [66]. Results showed that the most significant factor accounted for only 24.04% of the total variance, below the critical threshold of 50%. That suggests that common method variance in the data was acceptable and unlikely to impact the results significantly.

### 4.2 Construct validity

Cronbach’s α coefficients were used to assess the scales’ reliability. The results showed that all reliabilities of the scales, as shown in instruments, range from 0.76 to 0.95, indicating that they reached an acceptable level of internal consistency. Construct validity was evaluated through confirmatory factor analysis (CFA) of job demands, job resources, emotional labor strategies, and teaching efficacy. The results, as presented in Table 1, showed that the measurement model had the best data fit (c2 = 1046.04, df = 479, P<0.01, RMSEA = 0.069, IFI = 0.91, CFI = 0.91), and the other competing models have significantly worse fit indices.

**Table 1:** Construct validity analysis.

Model	*χ*2	*df*	χ2/*df*	IFI	TLI	CFI	REMSEA
Measurement model	1046.04	479	2.18	.91	.90	.91	.069
Three-factor model	1390.61	482	2.89	.84	.82	.84	.086
Two-factor model	1945.22	484	4.02	.74	.72	.74	.109
Single-factor model	2532.52	486	5.21	.64	.60	.64	.129

### 4.3 Descriptive statistics and correlation matrix

Table 2 presents this study’s mean and standard deviation of latent variables. The results show that title, degree, university category, and discipline are not correlated with other variables and, therefore, are not included in the table. Chang et al. (2011) concluded that above the mean indicates high levels of the corresponding construct [65], so as shown in Table 2, job demands (M = 4.02, SD = 0.66) are much higher than the mean 3, indicating that university teachers perceived high emotional demands from universities. Similarly, scores for deep acting (M = 3.82, SD = 0.67), naturally felt emotions (M = 3.81, SD = 0.80), instructional strategy (M = 3.87, SD = 0.61), class management (M = 4.01, SD = 0.60) and interpersonal relation (M = 4.10, SD = 0.63) are all above the mean, while the mean scores for job resources (M = 3.06, SD = 0.84) and surface acting (M = 3.02, SD = 0.86) are at the average level. As evidenced by the respective means, the mean scores for deep acting (M = 3.82) and naturally felt emotions (M = 3.81) exhibit significant elevation in comparison to surface acting (M = 3.02). The outcomes of the analysis of variance (ANOVA) indicate a noteworthy disparity among the three scores (F = 4.44, p<0.05). Subsequent to conducting Post Hoc Multiple Comparisons using the LSD method, the findings reveal a substantial decrease in surface acting compared to deep acting and naturally felt emotions. However, no significant distinction was observed between deep acting and naturally felt emotions. These results lend support to Hypothesis 1.

**Table 2:** Descriptive statistics and correlation matrix.

variables	1	2	3	4	5	6	7	8
1. Job demands								
2. Job resources	.063							
3. Surface acting	.126*	.242**						
4. Deep acting	.393**	.905**	.328**					
5. Naturally felt emotions	.225**	-.148**	-.395**	.226**				
6. Instructional strategy	.256**	.012	-.216**	.304**	.252**			
7. Class management	.232**	.001	-.255**	.226**	.234**	.804**		
8. Interpersonal relation	.252**	-.028	-.283**	.237**	.232**	.586**	.804**	
Mean (M)	4.02	3.06	3.02	3.82	3.81	3.87	4.01	4.10
Standard deviation (SD)	0.66	0.84	0.86	0.67	0.80	0.61	0.60	0.63

*Note*: *N* = 316, *p<0.05

**p<0.01.

Table 2 also presents the correlation matrix between the eight variables. The matrix shows that job demands are not significantly correlated with job resources. None of the correlations between job resources and teaching efficacy are significant. Teaching efficacy is significantly correlated with all the other variables. Furthermore, all the variables are significantly positively correlated with each other, except for surface acting, which is negatively correlated.

### 4.4 Hypothesis test

**Fig 2:**
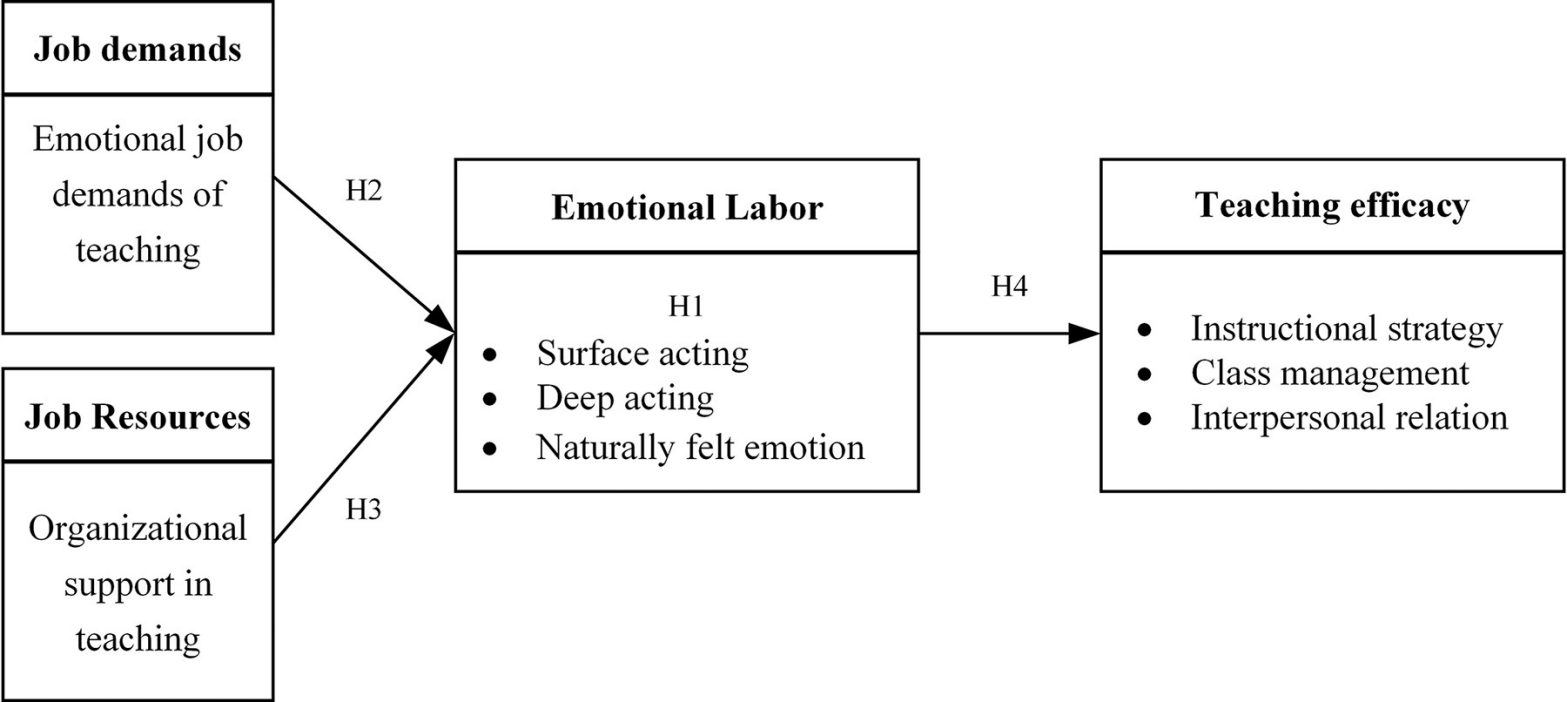
The SEM results of the relationships among variables. (*Note*: *N* = 316; *p<0.05, **p<0.01, ***p<0.001.).

Based on the hypothesized model shown in Fig 1, a structural equation modeling (SEM) was set up to test the relationships between emotional labor strategies, job demands, job resources, and teaching efficacy. The results of the SEM in this model are shown in Fig 2, indicating that the model achieved a good data fit. (c2 = 798.378, df = 415, p<0.01, c2/df = 1.92, RMSEA = 0.061, NNFI = 0.92, CFI = 0.93, IFI = 0.93, TLI = 0.92).

According to the results of the regression path analysis shown in Fig 2, the emotional job demands were positively associated with surface acting (β = 0.38, p<0.001; H2a), deep acting (β = 0.45, p<0.001; H2b) and naturally felt emotions (β = 0.23, p<0.05; H2c). Therefore, the H2 hypothesis was partially supported, where H2a and H2b were supported, and H2c was not supported. Job resources were positively associated with surface-acting (β = 0.26, p<0.001; H2a) and deep acting (β = 0.15, p<0.05; H2b), while there was no significant relationship with naturally felt emotions (β = -0.12, p>0.05; H2c). Therefore, H3b was supported; H3a and H3c were not supported, so the H3 hypothesis was partially supported.

Regarding the results between the three dimensions of emotional labor and the three dimensions of teaching efficacy, there were no significant relationships between surface acting and any of the three dimensions of teaching efficacy(H4a; instructional strategies β = -0.05, p>0.05; class management β = -0.09, p>0.05; interpersonal relation β = 0.02, p>0.05), while deep acting (H4b; instructional strategies β = 0.16, p<0.05; class management β = 0.16, p<0.05; interpersonal relation β = 0.17, p<0.05) and naturally felt emotions (H4c; instructional strategies β = 0.78, p<0.001; class management β = 0.89, p<0.001; interpersonal relation β = 0.97, p<0.001) were both positively associated with teaching efficacy in three dimensions. Therefore, H4b and H4c were supported, while H4a was not.

## 5. Discussions

### 5.1 University teachers prefer to show authenticity in teaching

This study diverges from prior research on teachers’ emotional labor by investigating the outcomes of the emotional labor of organizational concern. Specifically, the study examines the effects of different emotional labor strategies employed by university teachers on their teaching efficacy in areas such as instructional strategy, class management, and interpersonal relations. That is distinct from previous studies which measured teaching efficacy in terms of a general sense of personal accomplishment or specific operational behaviors in teaching [67]. The data collected in this study supports Hypothesis 1, which posits that university teachers tend to use deep acting and naturally felt emotions in teaching. In particular, expressing naturally felt emotions affect teaching efficacy, indicating that university teachers prefer these two strategies in emotional labor. These findings are consistent with previous research [7].

Deep acting and naturally felt emotions are usually perceived as authenticity [68]. Ozturka et al. conducted a study that found that individuals employed at universities for more than six years tended to experience moderate emotional labor characterized by sincere and authentic emotions [69]. University teachers typically refrain from pretense, as doing so is deemed unproductive. That may be because university students are highly attuned to recognizing emotions and expect their instructors to behave as mature adults. If surface acting is employed, it is likely to be perceived by students as insincere, thereby hindering future interactions and teaching effectiveness, as supported by the findings of this study. This research indicates that surface acting is not associated with teaching efficacy, underscoring teachers’ importance in employing appropriate strategies when dealing with students. Moreover, university teachers are typically characterized by a scientific spirit of factuality and a willingness to confront issues head-on. Pretense during teaching is not in keeping with these characteristics and is therefore deemed unnecessary.

### 5.2 Authenticity is effective affecting teaching efficacy

The results of this study supported hypotheses H4b and H4c but did not support H4a. Specifically, deep acting and naturally felt emotions were positively associated with teaching efficacy, including instructional strategies, class management, and interpersonal relations. At the same time, surface acting was not significantly related to any of these dimensions of teaching efficacy. These results indicated that the different emotional labor strategies employed by university teachers have varying impacts on their teaching efficacy, which is consistent with the findings of Xie et al. (2022) [62]. According to the job demands-resources model, deep acting and naturally felt emotions are beneficial processes that can reduce teachers’ emotional attrition and enhance their teaching efficacy. That is especially true for naturally felt emotions, which have a powerful effect on teaching efficacy. For Hypothesis H4a, based on previous literature [70], surface acting is considered an attrition process, and it is expected to have a negative predictive effect on teaching efficacy. However, the results of this study indicate no relationship between the two, a belief shared by prior scholars [68]. However, concerning university teachers, there may be several reasons for this discrepancy. Firstly, following the results of Hypothesis 1, it is evident that university teachers prefer deep acting and naturally felt emotions, with minimal utilization of surface acting, resulting in lower scores that are statistically insignificant. Secondly, these discrepancies may be due to participant differences and their understanding of outcome variables. Previous studies have primarily focused on teacher well-being, such as job burnout and satisfaction, and have primarily included primary and secondary school teachers. In contrast, teaching efficacy is a judgment related to professionalism. Most university teachers hold Ph.D. degrees and possess significant research experience, which can bolster their professional confidence even when they use surface acting.

### 5.3 Job demands and resources affect emotional labor in teaching

The job demands-resources model is valuable for understanding the factors influencing an individual’s emotional labor strategies. This study examined teachers’ perceived emotional demands of teaching and the resources provided by the university organization. The results revealed that emotional demands in teaching were positively associated with surface and deep acting, supporting hypotheses H2a and H2b and aligning with previous research by Yin et al. (2017) and Zheng et al. (2022) [5, 7]. However, the study also uncovered a positive correlation between job demands and naturally felt emotions among university teachers, contrary to the hypothesis proposed in H2c. These results suggest that demands on emotions in teaching prompt teachers to increase their expression of naturally felt emotions and use authenticity in their interactions with students. The study also found that job resources were positively associated with surface and deep acting and negatively related to naturally felt emotions, with H3b supported but not H3a and H3c. These results are consistent with the job demands-resources model [71], which suggests that organizational support is a job resource that motivates university teachers to use acting strategies when they do not have authentic emotions to cope with the emotional demands of teaching.

The research results indicated that H3a, contrary to the expected outcome, the provision of job resources did not reduce surface acting but instead increased it. The reasons for this phenomenon may be that job resources given by the organization based on job demands will motivate teachers to meet job demands, using surface acting and deep acting to increase the expression of emotions consistent with job demands, but will not lead to the expression of authentic emotions, results that are consistent with the findings of Zheng et al. (2022) and Yin (2015) [7, 72]. That is also consistent with the principle of reciprocity, but it depends on how teachers perceive the organizational support they receive; if it is treated as an external motivator and a complementary resource, it will reduce the resource depletion of employees by job demands and increase their motivation, who will regulate their external behavior according to the demands, which in turn will increase the occurrence of surface acting. Suppose job resources are treated as internal motivation. In that case, it motivates university teachers to change their perceptions from within their jobs and focus on self-growth and development, increasing the occurrence of deep acting. In addition, this may also be related to the fact that different teachers have different resources and perceptions of the organization’s job resources. For the same job resource, different teachers may interpret it as both a disguised job demand and a job resource, which produces a loss or gain effect on themselves. If they understand it as a job demand, they will use more surface acting; if they understand it as a job resource, they will use more deep acting [73].

Hypothesis H3c is not supported, which requires us to consider the relationship between job demands-resources and naturally felt emotions. Unlike surface acting and deep acting, naturally felt emotions do not require efforts to make cognitive changes in external behaviors and internal emotions. University teachers may use all available resources to meet the emotional job demands of teaching, including their own emotions, particularly when the authentic emotions are not aligned with the demands. However, according to the exchange theory, receiving resource support from the organization may lead to cognitive dissonance if teachers do not perform the behaviors required. Therefore, they may regulate this dissonance by acting and suppressing their genuine emotions, and as a result, the provision of job resources may not increase the expression of authentic emotions. Additionally, another reason is that university teachers have been continuously engaged in learning and working within the academic environment, which makes them relatively more straightforward and authentic compared to professionals in other fields. Consequently, they inherently prefer and are proficient in using genuine emotional expression, a notion supported by Hypothesis 1, suggesting that they will try to portray their authentic selves, regardless of whether the organization provides resource support.

## 6. Implications

### 6.1 Use authentic strategies in teaching

The study’s findings demonstrate the importance of authenticity in positively affecting teaching efficacy, specifically using deep acting and naturally felt emotions. Therefore, it is recommended that university teachers should thoughtfully select authentic strategies in their teaching practices. In particular, surface acting should be reduced while embracing authentic strategies, such as deep acting and naturally felt emotions, to enhance teaching efficacy and improve teaching effectiveness. By prioritizing authenticity in their instructional approaches, teachers can cultivate a more positive teaching experience for themselves and their students, leading to improved learning outcomes. Specifically, teachers can participate in training courses and workshops to systematically learn the application of authentic strategies in teaching. Meanwhile, they can further initiate research about the theories and applications of authentic strategies to realize the multidisciplinary integration of theory and practice [74]. Hence, adopting authentic teaching strategies is crucial in promoting effective teaching practices in Chinese universities.

### 6.2 Universities alleviate teachers’ emotional labor

Universities should pay more attention to the emotional state of teachers and provide resources to support them to alleviate their emotional labor. Universities’ support is constructive to staff work and performances [75, 76]. Instructional management usually emphasizes the outcomes of emotional labor while ignoring how university teachers regulate their emotions. According to the results of this study, universities place high demands on teachers’ emotions in teaching, and this type of job demand-focused management can significantly increase teachers’ emotional labor. The results showed that the expression of naturally felt emotions has improved, but surface acting and deep acting strategies have increased more noticeably. Over time, that could adversely affect teachers’ physical and mental health. Therefore, universities should exercise caution when imposing emotional demands on teachers. Even without such demands, teachers may have high expectations of themselves based on cultural norms; universities should instead provide resource support that mobilizes teachers’ intrinsic motivation and prioritizes their rights and well-being. It is crucial to make teachers aware of the relationship between job demands and their achievements and goals so that they can genuinely sense the care and support from the university. That, in turn, will help teachers adopt more authentic emotional strategies and reduce their use of acting behaviors in teaching.

### 6.3 Universities support teachers’ teaching

University teachers distinguish themselves from other groups of teachers with their loose organization, heightened self-awareness, and motivation to continually enhance their teaching skills. As such, overly detailed management demands are counterproductive for university teachers and may provoke a rebellious attitude. To support university teachers, universities should streamline administrative demands, reduce teacher burdens, and cultivate a harmonious environment that engenders trust and support. Specifically, universities can streamline the preparation of teaching materials, reduce the number of meetings per semester, and reduce non-essential non-teaching or research work. There is a coupling coordination relationship between university growth and teacher achievement; the two systems can achieve positive interactions and promote each other [77]. As Gardner note, when individuals feel trusted, they are more likely to show their true selves to others, which is advantageous for improving teaching effectiveness [78].

## 7. Conclusions

Based on the job demands-resources model, this study aimed to explore the relationships among emotional labor strategies, job demands of teaching, job resources, and teaching efficacy for university teachers, specifically, (1) we analyzed the current literature and constructed a research framework based on the job demands-resources model; (2) we collected data by employing a survey and used a structural equation modeling approach to test the relationships of the variables in the framework; (3) we proposed implication recommendations for university administrators based on the conclusions obtained.

We get the following key conclusions in this study. (1) In work, university teachers preferred to convey authenticity by expressing deep acting and naturally felt emotions, which were perceived as sincere. (2) Deep acting and naturally felt emotions had a significant positive effect on teaching efficacy, while surface acting had no significant effect. (3) Job demands as an organizational variable significantly influenced surface acting, deep acting, and naturally felt emotions positively. In contrast, job resources positively affected surface and deep acting but did not significantly affect naturally felt emotions. From the above, we found that authentic emotional labor strategies (deep acting and naturally felt emotions) are wise choices for university teachers.

This study has several innovative features. (1) we adopt the job demands-resources model to explore organizational variables’ effects on Chinese university teachers’ emotional labor. This study supports the applicability of the job demands-resources model to university settings and combines the job demands and resource approaches. The outcomes can furnish fresh evidence that contributes to a more comprehensive understanding of the effects of organizational factors on the emotional labor of college educators and their consequent job efficacy. (2) This study found significant differences between the emotional labor of university teachers and other service groups, with university teachers preferring authentic emotional labor strategies in working than other groups. (3) The results of this study provide straightforward ways for university administrators to improve the teaching and emotional labor strategies of university teachers; universities should pay more attention to teachers’ emotional states, provide resources to support them, and give more space and support to teachers in teaching.

While this study revealed significant results regarding the relationship between emotional labor and teaching efficacy, some limitations exist. Firstly, although the sample size of this study is representative, China has many universities, and results may vary across different types, regions, and majors. In future research, we will consider expanding the sample size to enhance the generalizability of the results. Secondly, the samples selected for this study were from Chinese universities. However, whether this is the case for teachers in universities in other countries is still being determined, and future comparative studies can be conducted via international cooperation.
